# Histidyl-Proline Diketopiperazine Isomers as Multipotent Anti-Alzheimer Drug Candidates

**DOI:** 10.3390/biom10050737

**Published:** 2020-05-09

**Authors:** Hasan Turkez, Ivana Cacciatore, Mehmet Enes Arslan, Erika Fornasari, Lisa Marinelli, Antonio Di Stefano, Adil Mardinoglu

**Affiliations:** 1 Department of Medical Biology, Faculty of Medicine, Atatürk University, 25240 Erzurum, Turkey; 2 Department of Pharmacy, University “G. d’Annunzio” of Chieti-Pescara, via dei Vestini 31, 66100 Chieti Scalo (CH), Italy; cacciatore@unich.it (I.C.); fornasari@unich.it (E.F.); marinelli@unich.it (L.M.); adistefano@unich.it (A.D.S.); 3 Department of Molecular Biology and Genetics, Faculty of Science, Erzurum Technical University, 25050 Erzurum, Turkey; enesiyte@gmail.com; 4 Science for Life Laboratory, KTH-Royal Institute of Technology, SE-17121 Stockholm, Sweden; 5 Centre for Host-Microbiome Interactions, Faculty of Dentistry, Oral & Craniofacial Sciences, King’s College London, London SE1 9RT, UK

**Keywords:** histidyl-proline diketopiperazine, Alzheimer’s disease, amyloid-beta 1-42, neuroprotection, novel therapeutics

## Abstract

Cyclic dipeptides administered by both parenteral and oral routes are suggested as promising candidates for the treatment of neurodegeneration-related pathologies. In this study, we tested Cyclo (His-Pro) isomers (cHP1-4) for their anti-Alzheimer potential using a differentiated human neuroblastoma cell line (SH-SY5Y) as an Alzheimer’s disease (AD) experimental model. The SH-SY5Y cell line was differentiated by the application of *all-trans* retinoic acid (RA) to obtain mature neuron-like cells. Amyloid-beta 1-42 (*Aβ_1-42_*) peptides, the main effector in AD, were administered to the differentiated cell cultures to constitute the in vitro disease model. Next, we performed cell viability analyses 3-(4,5-dimethylthiazol-2-yl)-2,5-diphenyltetrazolium bromide (MTT) and lactate dehydrogenase (LDH) release assays) to investigate the neuroprotective concentrations of cyclodipeptides using the in vitro AD model. We evaluated acetylcholinesterase (AChE), α- and β-secretase activities (TACE and BACE1), antioxidant potency, and apoptotic/necrotic properties and performed global gene expression analysis to understand the main mechanism behind the neuroprotective features of cHP1-4. Moreover, we conducted sister chromatid exchange (SCE), micronucleus (MN), and 8-hydroxy-2′-deoxyguanosine (8-OHdG) analyses to evaluate the genotoxic damage potential after applications with cHP1-4 on cultured human lymphocytes. Our results revealed that cHP1-4 isomers provide a different degree of neuroprotection against *Aβ_1-42_*-induced cell death on the in vitro AD model. The applications with cHP1-4 isomers altered the activity of AChE but not the activity of TACE and BACE1. Our analysis indicated that the cHP1-4 increased the total antioxidant capacity without altering total oxidative status levels in the cellular AD model and that cHP1-4 modulated the alterations of gene expressions by *Aβ_1-42_* exposure. We also observed that cHP1-4 exhibited noncytotoxic and non-genotoxic features in cultured human whole blood cells. In conclusion, cHP1-4 isomers, especially cHP4, have been explored as novel promising therapeutics against AD.

## 1. Introduction

In the last two decades, the peptide regulators or transmitters have received considerable attention in pharmacological and medicinal areas. The potential role of cyclo (His-Pro) (cHP), known as a cyclic dipeptide derived from the hydrolytic removal of the amino-terminal pyroglutamic acid residue of the hypothalamic thyrotropin-releasing hormone (TRH) in the prevention of neurodegeneration, has been widely studied. In fact, cHP provides conspicuous neuroprotection as the main substrate of organic cation transporters. Furthermore, it is shown that it can cross the brain–blood barrier and may alter inflammatory and stress responses by modifying the *Nrf2-NF-κB* signaling. In addition to these neuroprotection-related features, cHP has a nootropic nature; enhances cognitive function; modulates both necrotic and apoptotic neuronal cell death; lies behind of phosphorylation of the small heat shock proteins; ameliorates ER stress, excitotoxicity, and calcium overload; and supports antioxidant capacity. It has been also proposed that cHP interacts with a receptor dually coupled to stimulatory and inhibits G proteins. Due to its favorable features, cHP is considered a promising candidate for the treatment of neurodegenerative diseases like Alzheimer’s disease (AD) and amyotrofik lateral skleroz (ALS) [1,2,3,4].

Earlier studies revealed that there is a strong correlation between the protein levels in the spinal fluids and the age of patients. Interestingly, it is also determined that cHP levels decrease due to increased age of patients and then reaches to very low values in adulthood (300 pg/mL in adult age vs 1909 pg/mL in preterm babies). Again, not only cHP but also some of its derivatives are shown to prevent neuronal death induced by free radicals as well as calcium mobilization and traumatic injury [5,6,7,8]. Finally, it has been reported that the administration of cHP contributes to improvements of memory and to minimizing *Aβ_1-40_* and *Aβ_1-42_* protein levels and activates antioxidant responses in the brain tissues of the mice [9,10]. 

Based on these considerations, since much more has yet to be explored regarding the potential benefits provided from this compound in neurodegenerative diseases, we synthesized four stereoisomers of cHP (as cHP1-4) to perform in vitro studies and to assess their potential use as anti-Alzheimer agent (Figure S1). To evaluate its anti-Alzheimer potential, we treated differentiated human neuroblastoma SH-SY5Y cells with different concentrations of cHP1-4 and studied their responding viability against *Aβ_1-42_*-induced neurotoxicity by 3-(4,5-dimethylthiazol-2-yl)-2,5-diphenyltetrazolium bromide (MTT) and lactate dehydrogenase (LDH) release assays. To further investigate the neuroprotective effects of cHP1-4, we determined acetylcholinesterase (AChE) and *α*- and *β*-secretase (TACE and BACE1) activities along with total antioxidant capacity (TAC) and total oxidative status (TOS) levels. We also evaluated the effects of cHP1-4 on apoptotic and necrotic neuronal death using both flow cytometric and microscopic detection methods. We also investigated the molecular genetic responses to effective cHP isomers via PCR arrays in the in vitro AD model. The molecular genetic response studies contain 64 key genes in 10 different biological pathways including apoptosis; necrosis; DNA damage and repair; mitochondrial energy and fatty acid metabolisms; oxidative stress; and antioxidant, heat shock, and unfolded protein responses along with endoplasmic reticulum stress immunity and cytochrome p450s and phase I drug metabolisms. Finally, we evaluated the biosafety profiles of cHP1-4 using several cytotoxicity (MTT and LDH release assays) and genotoxicity (sister chromatid exchange (SCE) and micronucleus (MN) assays and 8-OH-dG level) endpoints on cultured human peripheral blood cells.

## 2. Material and Methods

### 2.1. Synthesis of cHP Isomers

We synthesized four stereoisomers of cHP to perform in vitro studies as previously reported [11]. 

### 2.2. Experimental Design and Treatments

The human SH-SY5Y neuroblastoma cell line was provided from the American Type Culture Collection (ATCC). SH-SY5Y cells were grown in DMEM:F12 (1:1) (Gibco^®^) media containing heat-inactivated fetal bovine serum (FBS, 10%) (Sigma-Aldrich^®^, USA) and penicillin/streptomycin (Sigma-Aldrich^®^, 1%) at 37 °C and 5% CO_2_ up to cells reach confluence. SH-SY5Y cells were harvested using trypsin/ethylenediaminetetraacetic acid (EDTA) (Sigma-Aldrich^®^) solution and transferred to 24-well plates. For the differentiation process, 10 μM retinoic acid (RA, Sigma-Aldrich^®^) was applied to the media and incubated for six days. After the incubation period, this media was changed into serum-free media containing RA (10 μM) and brain-derived neurotrophic factor (BDNF, 25 nM, Promega^®^). Differentiated cells were investigated under the inverted microscope (Olympus CKX41, USA), and cellular differentiations were confirmed via flow cytometric cell cycle analysis [12]. Commercially available *Aβ_1-42_* peptides (C_203_H_311_N_55_O_60_S, Sigma-Aldrich^®^) were firstly dissolved in dimethyl sulfoxide (DMSO) to prevent peptide aggregations and then integrated into the cell culture in the desired concentration (20 μM). When the *Aβ_1-42_* peptides were added to the cell culture with DMSO, the final concentration was calculated as 1% for DMSO in each well to prevent any solvent toxicity. Differentiated cells were treated with selected concentrations of cHP1-4 (0.1, 1, 10, 25, 50, and 100 μM) and *Aβ_1-42_* (20 μM) (Sigma-Aldrich^®^) for 24 h (*n* = 5). 

### 2.3. Determination of Cellular Viability 

Cell viability was measured using MTT and LDH release assays. For MTT assay, briefly, the neuron-like SH-SY5Y cells were seeded into 24-well plates with 5000 cells per well. The cells were exposed to cHP1-4 at different concentrations (0 to 100 µM) for 24 h at 37 °C. At the end of the incubation period, MTT solution (170 μL) was added into each well. Plates were incubated for 4 h at 37 °C, and then, plates were centrifuged. The formazan crystals were dissolved in 200 μL dimethyl sulfoxide (DMSO) (Sigma-Aldrich^®^). The absorbance was measured at 570 nm using a microplate reader (BioTek^®^). Cell viability was calculated as a percentage relative to the untreated control value [13,14,15,16,17]. 

Commercial LDH cytotoxicity assay kit (Cayman Chemical Company^®^) was used according to the manufacturer’s recommendations. Concisely, after the treatment with cHP1-4, the cells in 24-well plate were centrifuged at 1000 *g* for 5 min and supernatants were obtained; 100 µL supernatant and 100 µL of the reaction mixture were placed to a fresh plate and incubated at for 40 min room temperature. The LDH release was measured using a microplate reader (at 490 nm) and presented as a percentage (%) of total LDH release, which was accorded to the equation % LDH released = (LDH amount in medium/total LDH amount) × 100 [18]. 

### 2.4. Determination of AChE, TACE, and BACE1 Activities

The activities of AChE, TACE, and BACE1 on the AD in vitro model were measured by commercially available kits. For determinations of AChE activities, the colorimetric acetylcholinesterase assay kit from Abcam^®^ was used due to the manufacturer’s guide. Likewise, TACE and BACE1 activities detected via using fluorometric tumor necrosis factor-α converting enzyme (TACE) and β-secretase (BACE1) activity detection kits (Sigma-Aldrich ^(R)^) acted upon to the recommended protocol by the provider.

### 2.5. Determination of TAC and TOS Levels 

The TAC and TOS levels were determined using commercially available kits (Rel Assay^(R)^, Turkey) on samples obtained from cHP1-4 treated and untreated cultures. Ascorbic acid ( C_6_H_8_O_6_, 10 μM) and hydrogen peroxide (H_2_O_2_, 25 µM) (Sigma-Aldrich^®^) were used as positive control groups (Control^+^) for TAC and TOS analysis, respectively [19].

### 2.6. Determination of Apoptosis and Necrosis by Fluorescence Microscopy and Flow Cytometry

The procedure of Hoechst 33258 staining was performed to evaluate apoptotic cells morphologically. For this, the untreated and treated neuron-like cells were fixed with 4% paraformaldehyde in phosphate buffered saline (PBS) at 4 °C for 20 min. Then, the cells were washed twice with PBS and stained with fluorescent Hoechst 33258 dye (1 mM, Sigma-Aldrich^®^). Finally, cells were analyzed and photographed under the fluorescence microscopy (Leica^®^ DM IL LED) [20]. 

The rates of viable, apoptotic, and necrotic cells were also determined by the Annexin V-Fluorescein isothiocyanate (FITC) apoptosis detection kit I (BD Pharmingen^®^). After incubation, 5 µL of Annexin V-FITC and 5 µL of propidium iodide (PI, 50 µg/ml) were added to the cultures and incubated in the dark for 10 min. Then, cells were fixed with 4% paraformaldehyde in phosphate-buffered saline at 4 °C for 20 min and were analyzed by a flow cytometer (CyFlow Cube 6, Partec^®^). Four cell populations types were determined with the Annexin V-FITC kit: (1) viable cells which were annexin negative and PI negative, (2) early apoptotic cells which were annexin positive and PI negative, (3) late apoptotic/necrotic cells which were annexin positive and PI positive, and (4) necrotic cells which were annexin negative and PI positive [21,22].

### 2.7. Determination of the Molecular Genetic Basis of Neuroprotection by cHP1-4 

Total RNA was isolated via using the PureLink^®^ RNA Mini Kit (Invitrogen^®^) due to protocol as suggested by the manufacturer. RNA yield and quality were determined at wavelengths of 260 and 280 nm using a plate reader (Multiscan, Thermo Labsystems^®^). Isolated total RNA was reverse transcribed into cDNA using commercially available High-Capacity cDNA Reverse Transcription kit (Applied Biosystems^®^). Then, total cDNA was used to perform expression analysis by RT^2^ Profiler PCR Array Human Molecular Pathway Finder (Qiagen^®^). For performing PCR array, SYBR Green PCR Master Mix (1150 μL, Applied Biosystems^®^), cDNA synthesis reaction (102 μL), and RNase-free water (1048 μL) were mixed. PCR components mix (20 μL) was added into each well. The reaction was started with an initial denaturation step at 95 °C for 10 min and then 40 cycles of 15 s at 95 °C and 30 s at 60 °C in real-time cycler (Qiagen Rotor-Gene Q^®^). The threshold cycle (CT) for each well was determined via the real-time cycler software. CT values handled analyzing the expression fold changes of target genes by web-based PCR Array Data Analysis Software (The GeneGlobe Data Analysis Center-Qiagen^®^). Total 64 key genes were studied relevant with 10 different pathways involving apoptosis (*AKT1, BCL2, BCL2L1, CASP8, CASP9,* and *FASLG*), necrosis (*CYLD, GRB2, MAG, PARP2, PVR,* and *TXNL4B),* DNA damage and repair (*ATM, BRCA1, CDKN1A, ERCC2, MDM2,* and *RAD51)* mitochondrial energy metabolism (*ARRDC3, ASB1, CYB561D1, DNAJB1, HSPA1A, HSPA1B,* and *SLC25A25),* fatty acid metabolism (*ACADM, ACADVL, ACOX1, CPT1A, CPT1B,* and *CPT2*), oxidative stress and antioxidant response (*AKR1C2, FHL2, GCLM, HMOX1, NCOA7, NQO1,* and *SLC7A11),* heat shock response (*HSF1, HSP90B1, HSPA4, HSPA8, HSPB1,* and *HSPD1),* endoplasmic reticulum stress-unfolded protein response (*ADM2, ASNS, DNAJB9, HERPUD1, INHBE, TRIB3,* and *UHRF1),* cytochrome P450s-phase I drug metabolism (*CYP1A2, CYP2B6, CYP2C19, CYP2C9,* and *CYP2D6),* and immunotoxicity (*ADH1C, F2, HPX, LYZ, METAP2, MKI67, NR5A2,* and *TRIM10).*

### 2.8. Determination of Cytotoxic and Genotoxic Potentials of cHP1-4

Peripheral human whole blood cells were used to assess the biosafety of cHP isomers. The whole blood cultures were set up as described previously [23]. After obtaining written informed consent forms from the volunteers, the blood samples were drawn from five healthy, nonsmoking males aged 28–32 years, with no recent history of exposure to mutagens as occupationally. The heparinized blood (0.7 mL) was cultured in 7 mL of culture medium (Gibco^®^) with 4.0 mg/mL of phytohemagglutinin (Sigma Aldrich^®^). The cHP isomers were exposed to the cultures just before the incubation. Control^−^ and control^+^ (10^−7^ M of mitomycin-C, MMC, Sigma^®^) groups were also set up. To evaluate cytotoxic and genotoxic damage potentials of the dipeptides, MTT, LDH release, sister chromatid exchange (SCE), micronucleus (MN), and 8-hydroxy-2′-deoxyguanosine (8-OH-dG) tests were carried out.

For scoring SCE formations, 5-bromo-2-deoxyuridine (BrdU, Sigma Aldrich^®^) was applied at culture initiation, and 70 h and 15 min after the inception of the incubations, n-diacetyl-n-methylcolchicine (Sigma Aldrich^®^) was added into the culture tubes. After hypotonic treatment (0.075 M KCl), fixation, centrifugation, and resuspension steps, the cell suspension was dropped onto icy microscopic slides. Then, the slides were dried and aged for four days. To visualize SCEs, the fluorescence plus Giemsa (FPG) procedure was followed. For each experimental group, well-spread 30 metaphase plaques (containing 42–46 chromosomes per cell) were examined and the obtained values were calculated as SCEs/cell [24].

The MN assay was practiced with the addition of cytochalasin B (6 μg/mL, Sigma^®^) after 44 h of culture and then incubated for an additional 28 h. Following the incubation period for 72 h, the cells were washed and suspended in Roswell Park Memorial Institute (RPMI) 1640 medium containing 4% FBS. The cells were fixed on the slides and stained with Giemsa solution (Sigma^®^). The slides were scored at 400× magnification using a bright-field microscope (Olympus). At least 2000 binucleated cells (BNCs) were scored per slide, and the results were presented as the number of MN/1000 BNCs. The MN criteria for scoring were applied as previously reported [25]. Besides, cytostatic potentials of cHP isomers were evaluated using the nuclear division index (NDI). 

To assess NDI rates, 500 cells per treatment group were analyzed for the presence of one, two, or more than two nuclei and the nuclear division index (NDI) was calculated as NDI = (1N + (2 × 2N) + (4 × >2N))/C, where 1N represents the number of cells with one nucleus, 2N is with two nuclei, >2N is with more than two nuclei, and C represents the total number scored cells [26].

8-hydroxy-2′-deoxyguanosine assay kits (Cayman Chemical^®^) were used for the determination of 8-OH-dG levels. All procedures were performed according to the provider’s manual. 

### 2.9. Statistical Analysis

The obtained data are presented as mean ± S.D. from five in vitro experiments. In the statistical analysis of the data, one-way analysis of variance (ANOVA) and Duncan’s test were performed according to the statistical program SPSS software (version 20.0, SPSS, Chicago, IL, USA). *p*-value of less than 0.05 was considered statistically significant.

## 3. Results

### 3.1. cHP Isomers Provided a Different Degree of Neuroprotection against Aβ_1-42_ Induced Cell Death in In Vitro AD Model

We differentiated human SH-SY5Y neuroblastoma cells to neuron-like cells by using RA and BDNF (Figure S2) and performed the cell cycle test to confirm cellular differentiation by using flow cytometry analysis. According to cell cycle phases, we found that total cells in the S phase are significantly decreased and increased in the G1 phase in differentiated SH-SY5Y cells compared to undifferentiated cells (Table S1). After exposure to 20 μM *Aβ_1-42_*_,_ we measured the viability rates in differentiated SH-SY5Y cells as 50.83% and 48.94% in MTT and LDH release assays, respectively. On the contrary, exposure of differentiated SH-SY5Y cells to different concentrations of cHP1-4 (1–100 μM) alone for 24 h did not alter the viability. Moreover, *Aβ_1-42_*-induced neurotoxicity was attenuated by co-treatment of cells with cHP1-4. We found that the attenuation effects of cHPs depend on concentration and isomer type-dependent manners in MTT assay (Table 1). cHP4 at 100 μM concentration almost neutralized *Aβ_1-42_*-induced cell death compared to the control^−^ group. To further reveal the neuroprotective functions of cHP1-4, we determined the release of LDH in the presence of *Aβ_1-42_*. When differentiated SH-SY5Y cells were treated with cHP1-4, we observed that the level of LDH is not change compared to the untreated group, showing their nontoxic feature. Furthermore, cell cultures co-treated with cHP1-4 (from 0.1 to 100 μM) led to a decrease in the amounts of LDH leakage. We also observed that the findings of the cell viability analysis revealed the protective effect of cHP1-4 against *Aβ_1-42_*-induced neurotoxicity. In fact, the increasing order in effectiveness of tested dipeptides against neurotoxicity by *Aβ_1-42_* was established as cHP1 < cHP2 < cHP3 < cHP4 (Table 1).

### 3.2. The Applications with cHP Isomers Altered the Activity of AChE but not the Activity of TACE and BACE1

**Figure 1:**
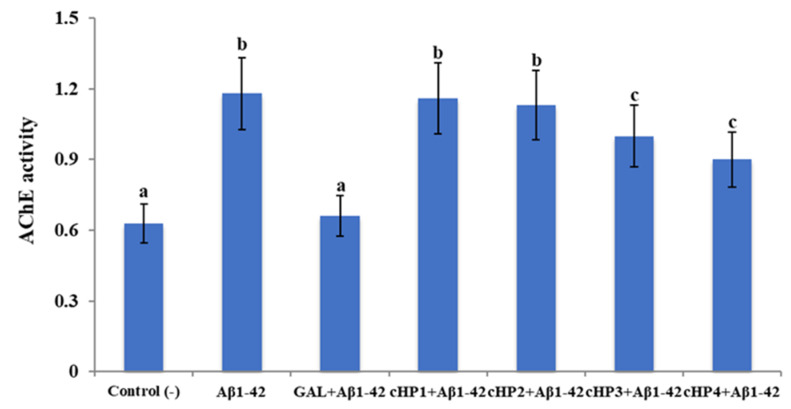
The in vitro effects of cHP1-4 applications (at 100 µM) on Aβ1-42-induced acetylcholinesterase (AChE) activity (as mU/ml) on a cellular Alzheimer’s disease (AD) model: Means with a different letter are significantly different from each other at the level of *p* < 0.05. Aβ1-42 vs. control (−): *p* < 0.0001, Aβ1-42 vs. GAL + Aβ1-42: *p* = 0.0002, Aβ1-42 vs. cHP1 + Aβ1-42: *p* = 0.9997, Aβ1-42 vs. cHP2 + Aβ1-42: *p* = 0.9926, Aβ1-42 vs. cHP3 + Aβ1-42: *p* = 0.0490, Aβ1-42 vs. cHP4 + Aβ1-42: *p* = 0.0430, and control (−) vs. GAL + Aβ1-42: *p* > 0.9999.

Our analysis indicated that the exposure to Aβ1-42 (at 20 µM) caused a statistically significant (*p* < 0.05) increase in the rate of 87.30% in baseline AChE activity compared to the untreated control group. We found that the treatments with cHP1-4 alone do not alter the AChE activity in the in vitro AD model and that their relatively lower concentrations (0.1–50 µM) are ineffective against *Aβ*-induced AChE activity (data not shown). Only the highest cHP concentrations (100 µM) provided a slight amelioration of AChE activity. We observed that cHP3 and cHP4 are determined as the most significant ones among tested cHP isomers and that the treatment with galantamine (GAL, as a positive control) strongly modulates *Aβ*-induced AChE activity (Figure 1). 

**Figure 2:**
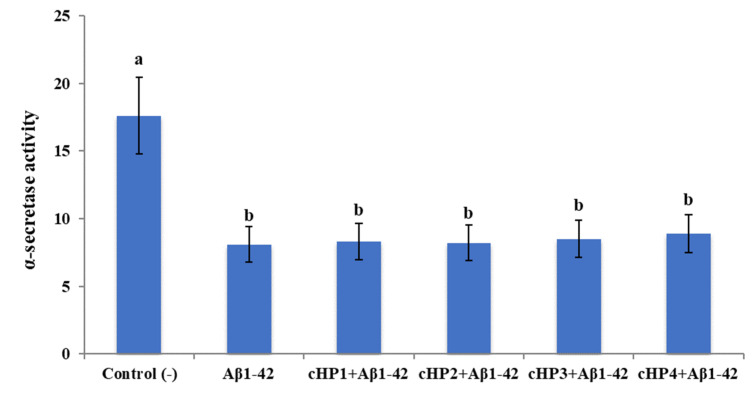
The in vitro effects of the applications with cHP1-4 (at 100 µM) on Aβ1-42-induced α-secretase activity (as fluorescence intensity/100 µg protein) on a cellular AD model: Means with a different letter are significantly different from each other at the level of *p* < 0.05. Aβ1-42 vs. control (−): *p* < 0.0001, Aβ1-42 vs. cHP1 + Aβ1-42: *p* = 0.9997, Aβ1-42 vs. cHP2 + Aβ1-42: *p* = 0.9999, Aβ1-42 vs. cHP3 + Aβ1-42: *p* = 0.9958, and Aβ1-42 vs. cHP4 + Aβ1-42: *p* = 0.9236.

**Figure 3:**
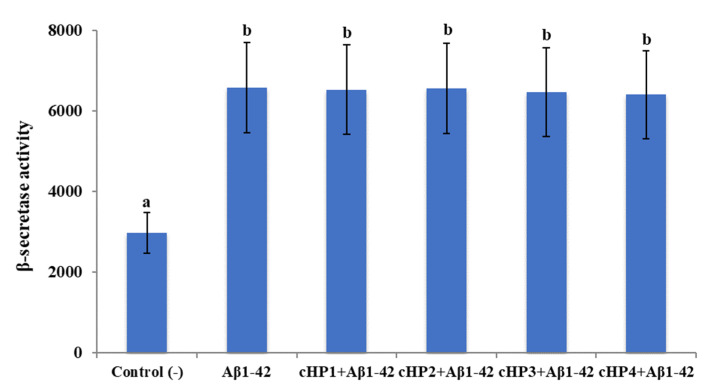
The in vitro effects of the applications with cHP1-4 (at 100 µM) on Aβ1-42-induced β-secretase activity (as fluorescence intensity/100 µg protein) on a cellular AD model: Means with a different letter are significantly different from each other at the level of *p* < 0.05. Aβ1-42 vs. control (−): *p* < 0.0001, Aβ1-42 vs. cHP1 + Aβ1-42: *p* > 0.9999, Aβ1-42 vs. cHP2 + Aβ1-42: *p* > 0.9999, Aβ1-42 vs. cHP3 + Aβ1-42: *p* = 0.9997, and Aβ1-42 vs. cHP4 + Aβ1-42: *p* = 0.9986.

We found that exposure to Aβ1-42 led to a significant increase of β-secretase activity (about 2.2-fold) and a significant decrease of α-secretase activity (about 2.1-fold). However, the treatments with certain concentrations of cHP1-4 alone did not change the activities of α- and β-secretases in differentiated SH-SY5Y cell cultures (data not shown). Also, the co-treatments of the isomers with *Aβ_1-42_*_,_ even at their highest application concentrations (100 µM), were not able to modulate *Aβ_1-42_*-induced activity changings (Figure 2 and Figure 3). 

### 3.3. cHP1-4 Supported Total Antioxidant Capacity without Altering Total Oxidative Status Levels in the Cellular AD Model

We measured the TAC and TOS levels after treating the in vitro AD model with cHP1-4 (Table 2). Our results indicated that all tested dipeptides induce antioxidant capacity more or less without altering TOS levels. In fact, we found that treatment with cHP1, cHP2, cHP3, and cHP4 led to increases of TAC levels by 62.5%, 35.4%, 33.3%, 58.3%, and 85.4%, respectively. Therefore, the decreasing order of effectiveness of cHP1-4 in inducing antioxidant capacity was cHP4 > cHP3 > cHP1 > cHP2. 

On the other hand, our results indicated that *Aβ_1-42_* exposure caused significant (*p* < 0.05) decreases with TAC and increases in TOS levels in vitro. Next, the exposure to 20 µM *Aβ_1-42_* reduced the TAC level approximately by 84.6% and elevated the TOS level approximately by 138.1%. Contrariwise, cHP1, cHP2, cHP3, and cHP4 (at 100 µM) alleviated decreased TAC levels by *Aβ_1-42_* by 53.8%, 23.1%, 23.1%, 46.1%, and 76.9%, respectively. Similarly, treatments with cHP1, cHP2, cHP3, and cHP4 (at 100 µM) modulated increased TOS levels by *Aβ_1-42_* by 39.2%, 23.5%, 21.6%, 29.4%, and 47.1%, respectively. Consequently, cHP4 was established as the most effective dipeptide in modulating cellular oxidative damages by *Aβ_1-42_*-exposure in the in vitro AD model (Table 3). 

### 3.4. cHP4 Provided In Vitro Protection to Neuron-Like Cells against Apoptotic and Necrotic Effects by Aβ_1-42_ in Cellular AD Model 

**Figure 4:**
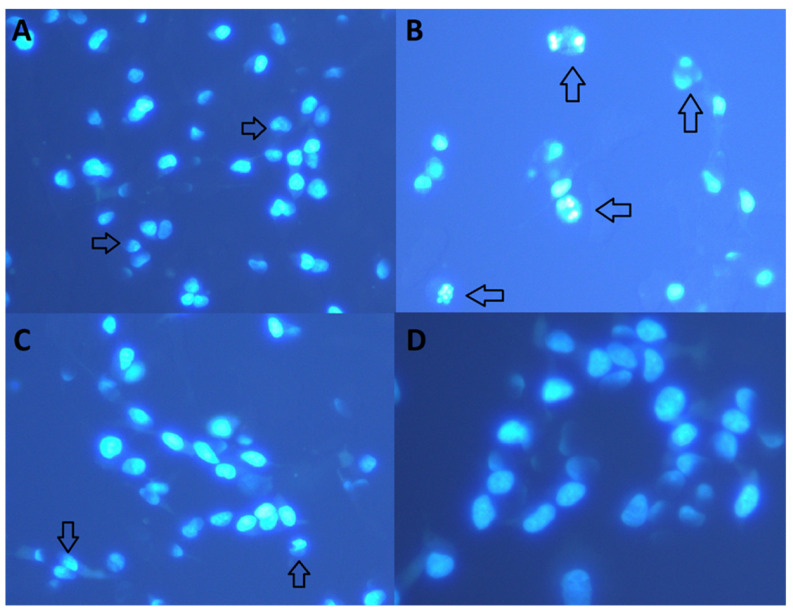
The effect of cHP4 on apoptotic and necrotic cell death in a cellular AD model (Hoechst 33258): (**A**) control (−) group, (**B**) Aβ1-42 (20 µM) + Aβ1-42, (**C**) cHP4 (50 µM) + Aβ1-42, and (**D**) cHP4 (100 µM) + Aβ1-42. Arrows show necrotic cells with damaged chromosomal structure.

Staining with Hoechst 33258 and visual analysis established that treatment with different cHP4 concentrations prevented cell death by *Aβ_1-42_* (Figure 4A–D). Our analysis indicated that *Aβ_1-42_*-induced neurotoxicity involved the necrotic pathway and the apoptotic pathway. The cHP1-4 provided a different degree of protection against *Aβ_1-42_*-induced neurotoxicity. Ultimately, the decreasing order of effectiveness in preventing cell death against *Aβ_1-42_*-induced cell death via dipeptides was determined as cHP4 > cHP3 > cHP1 > cHP2. 

**Figure 5:**
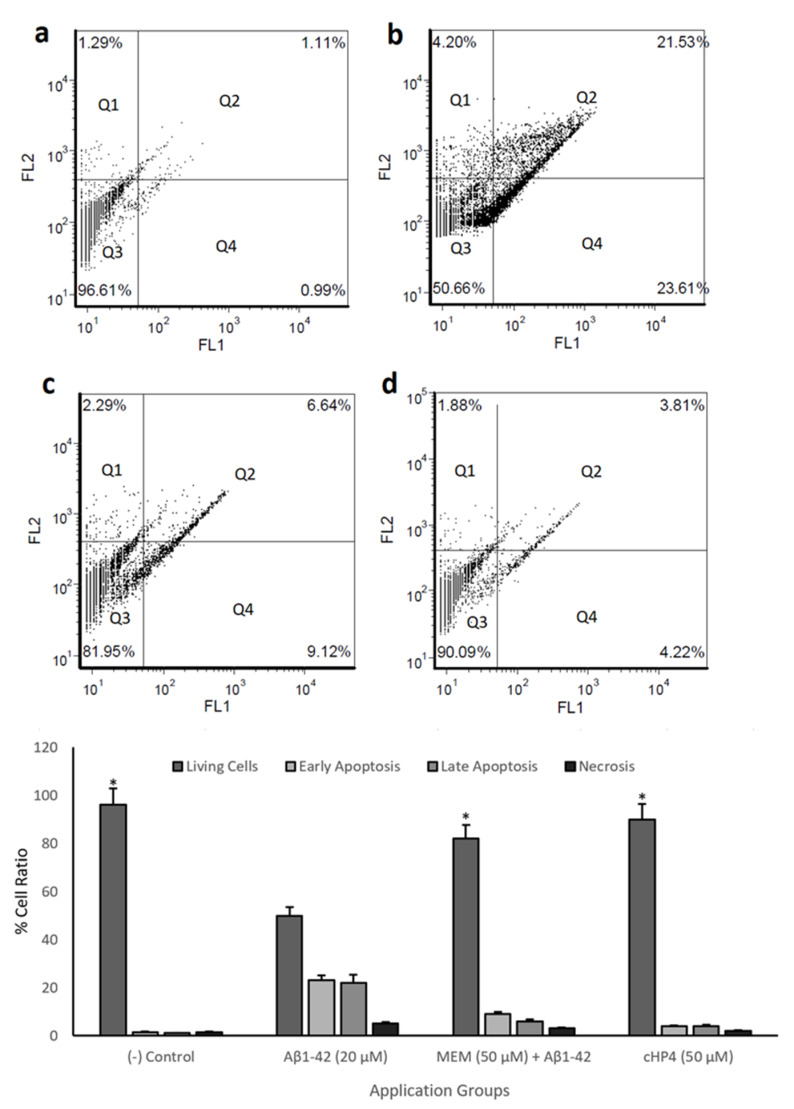
Flow cytometric analysis of Annexin V-FITC/PI double-labeled differentiated SH-SY5Y cells: (**a**) (−) control, (**b**) Aβ1-42 (20 µM), (**c**) memantine hydrochloride (MEM) (50 µM) + Aβ1-42, and (**d**) cHP4 (50 µM) + Aβ1-42. Quadrants were shown as Q1: necrosis, Q2: late apoptosis, Q3: living cells, and Q4: early apoptosis. The symbol (*) represents statistically significant difference (*p* < 0.01) of viable cell ratios compared to Aβ1-42 application. GraphPad Prism 7, Anova: Dunnett’s multiple comparison test was used to calculate the values: Aβ1-42 vs. (−) control: *p* < 0.0001, Aβ1-42 vs. MEM: *p* = 0.0002, and Aβ1-42 vs. GPE3: *p* < 0.0001.

We performed cell viability and biochemical and morphological analysis and identified cHP4 as the most protective dipeptide against *Aβ_1-42_*-induced neurotoxicity. We also performed flow cytometric analysis with cHP4 and observed the neuroprotective potential of cHP4 against apoptosis and necrosis by *Aβ_1-42_*-exposure compared to memantine hydrochloride (MEM) using flow cytometry. Our analysis revealed that *Aβ_1-42_* exposure led to a significant (*p* < 0.05) cell death rate particularly via early (Q2) and late apoptosis (Q4). However, cHP4 and MEM led to decreases in *Aβ_1-42_*-induced apoptotic cell percentage by 37.11% and 29.38%, respectively. Finally, we evaluated if cHP4 exhibited more potential on the protection of the human neuron-like cells from *Aβ_1-42_*-induced necrosis and apoptosis than MEM and presented the results (Figure 5A–D).

### 3.5. cHP4 Modulated the Alterations of Gene Expressions by Aβ_1-42_ Exposure 

To reveal the molecular mechanisms underlying neuroprotection by cHP4, we performed gene expression analysis using RT^2^ Profiler PCR Arrays. We measured the expression of 64 key genes involved in 10 different pathways. We found that the expression of the genes including *CASP8, CASP9, ERCC1, HSPA1A*, and *PARP2 is* significantly increased whereas the expressions of the genes including *ACADVL, ADM2, BCL2, BCL2L1, CYP2D6, DNAJB9, DNAJB9, FASLG, METAP2, SLC7A11,* and *UHRF1* is significantly decreased after exposure to *Aβ_1-42_*. Our results also showed that application with cHP4 alone did not alter the expression profiling of these genes. Furthermore, cHP4 alleviated the actualized expressional alterations of these genes by *Aβ_1-42_* exposure (Table 4). 

### 3.6. cHP4 Exhibited Noncytotoxic and Non-Genotoxic Features in Cultured Human Whole Blood Cells

**Figure 6:**
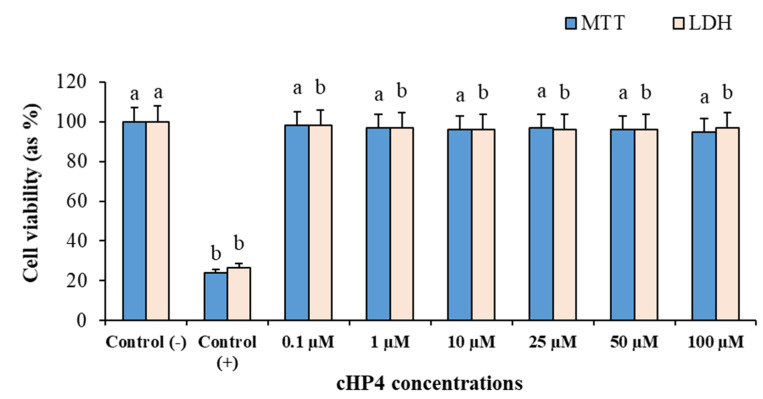
The cytotoxic responses to cHP4 in cultured human blood cells: Means with a different letter are significantly different from each other at the level of *p* < 0.05.

To determine the cytotoxic potential of cHP4 applications (0.1 to 100 µM), we performed MTT and LDH release assays using cultured human whole blood cells. The results of cytotoxicity testing showed that Triton-X (%1, control (+)) led to a significant decrease in the cell viability rates compared to the control (−) group. We found that the cell viability rates in MTT and LDH release assays are 76.1% and 73.6%, respectively. On the contrary, the applications with cHP4 concentrations did not alter the cell viability rates compared to the control (−) group (Figure 6). In brief, the results of MTT and LDH release analysis in human blood cells indicated the non-cytotoxic nature of cHP. 

**Figure 7:**
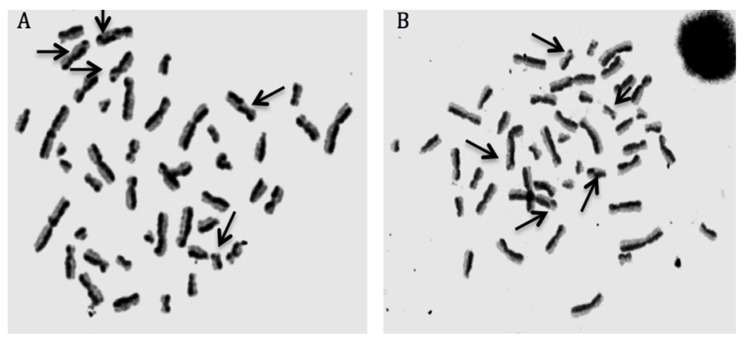
Sample metaphases from (**A**) mitomycin-C (MMC)-treated (as positive control) and (**B**) cHP4 (100 µM)-treated human lymphocytes.

**Figure 8:**
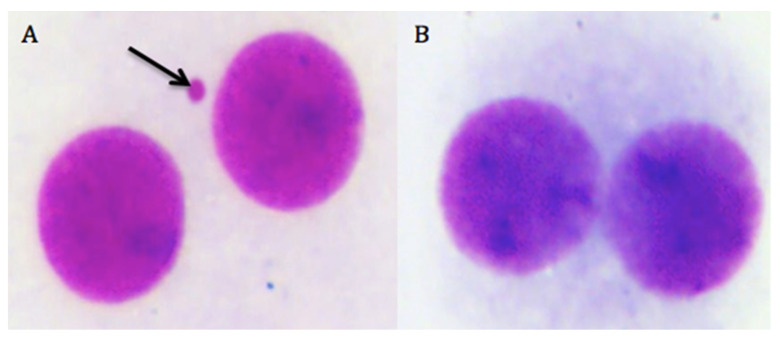
The photomicrograph of binucleated cells from (**A**) the positive control group as MMC and (**B**) cHP4 (100 µM)-treated human lymphocytes (arrow indicates the formation of MN).

**Figure 9:**
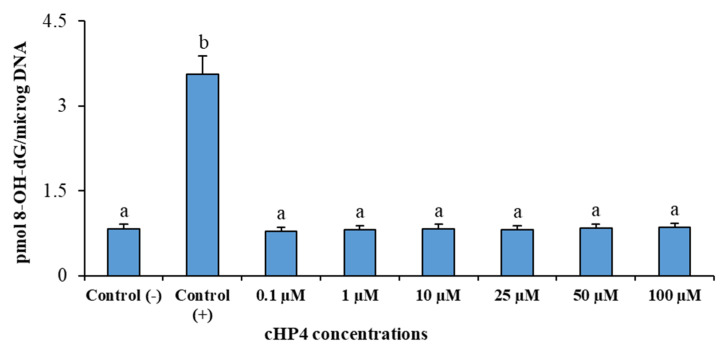
The determined 8-oxo-2-deoxyguanosine (8-OHdG) levels in human whole blood cultures treated with different cHP4 concentrations for 72 h: Means with a different letter are significantly different from each other at the level of *p* < 0.05.

We presented the results of genotoxicity testing and NDI analysis after treatment with different concentrations (0.1, 1, 10, 25, 50, and 100 µM) of cHP4 in cultured human lymphocytes at Table 5. We observed that the cHP4 applications at all tested concentrations did not induce SCE or MN formations compared to control (−) (Figure 7 and Figure 8). As presented in Table 5, we found that MMC (as control (+)) caused a statistically significant decrease of NDI compared to untreated cultures but that cHP4 at all tested concentrations did not change the observed rates of NDI negatively. Therefore, cHP4 could be considered as non-cytostatic even at the applied highest concentration (100 µM). Besides, 8-OH-dG, a reliable oxidative DNA damage marker, was also determined in human whole blood cultures. The detected levels of 8-OH-dG are shown in Figure 9. It is determined that MMC (at 10^−7^M) caused a significant elevation of 8-OH-dG levels (about 4.3 folds) in cultured blood cells for 72 h. On the contrary, after treatment with cHP4 at concentrations between 0.1 and 100 µM, the determined 8-OH-dG levels were not found to be statistically different from untreated cultures. Consequently, the present results from SCE, MN, NDI, and 8-OH-dG testing revealed that cHP4 exhibited non-genotoxic property on human blood cells. 

## 4. Discussion

There are many studies which are investigating the SH-SY5Y neuroblastoma differentiation into mature neuron-like cell culture, facilitating neurobiology, and studying diseases like Alzheimer’s disease [27,28]. Recently, SH-SY5Y cell cultures are used in disease studies extensively as routine for in vitro neuro-disease projects. Protein expressions and morphological properties of the differentiated neuroblastoma cell line was investigated extensively and presented in different studies [13,14]. Thus, differentiation was monitored via microscopic technique, and flow cytometric cell cycle analysis was used for confirmation in our study. Also, the application of beta-amyloid peptides to the differentiated SH-SY5Y cell culture have been used as an alternative in vitro disease model for Alzheimer’s disease [29,30,31]. Our analysis indicated that *Aβ_1-42_* treatment led to a significant (*p* < 0.05) necrotic death rate in differentiated SH-SY5Y cell cultures. In accordance with this finding, it is suggested that *Aβ_1-42_*_-_induced cell death is related to necrosis and mediated by cell-to-cell interaction and via apoptosis [32,33]. According to the investigations, *all-trans* RA application coupled with brain derivative neurotrophic factor (BDNF) can fully differentiate the SH-SY5Y neuroblastoma cell line into human neuron-like cells and cells were arrested in the G1 cell cycle phase. On the other hand, RA application to the neuroblastoma cell line alone is shown to result in a high apoptotic cell death case due to the increased caspase activity. Also, integration of BDNF into the differentiation procedure is investigated to decrease the caspase activity and nearly no apoptosis. Moreover, differentiated cells were investigated to have highly phosphorylated tau proteins which is suitable for constituting AD cell culture model [12,13]. After the treatment with cHP1-4, the viable cell rates were elevated compared to only *Aβ_1-42_* treated cultures. Moreover, the established neuroprotectivity was related to the dipeptide type and applied concentration. cHP4 was determined as the most effective one for decreasing cell death induced by *Aβ_1-42_* among other cHP isomers. In parallel to this finding, several in vivo and in vitro findings revealed that cHP and its isomers provided neuroprotection. Furthermore, cHP was found to be effective in reducing *Aβ_1-42_* and *Aβ_1-40_* in the AD transgenic mouse model. However, the mechanisms that lie behind the neuroprotective action of cHP1-4 are still unknown [34].

Due to the results of MTT and LDH release assays as well as morphological and flow cytometric analysis, we determined that the applications with cHP1-4 provided neuroprotection against toxicity by *Aβ_1-42_* via preventing mainly necrosis and apoptosis generation. As a matter of fact, after the treatment with *Aβ_1-42_*, the expressions of the necrosis and apoptosis pathway-related genes including *PARP2, FASLG, CASP8,* and *CASP9* are significantly increased while the expressions of *BCL2* and *BCL2L1* genes are significantly decreased. Besides, a slight increase of *AKT1* expression was also determined after *Aβ_1-42_* exposure to differentiated SH-SY5Y cells (Table 4). A previous study revealed that *Aβ*-induced pro-apoptotic proteins lead to increases of *PARP1* activity in SH-SY5Y cells [35]. The elevated expressions of *PARP1-4* were determined under oxidative stress conditions and induced necrosis via the energy crisis [36]. The expression of *FASLG* was remarkably increased in association with caspase activation and neuronal apoptosis [37]. Likewise, *Aβ_1-42_* was shown to activate *CASP8* and *CASP9* [33]. It was also reported that *both Aβ_1-42_* and *Aβ_25-35_* slightly elevated *AKT1* expression in SH-SY5Y cell cultures [38]. On the other hand, exposing cells to *Aβ* caused downregulation of the expression for the antiapoptotic *BCL2* and *BCL2L1* genes in the PC12 cell line [39]. At this point, the application with cHP4 modulated these observed negative expressional alterations by *Aβ_1-42_* exposure. To sum up, cHP4 particularly ameliorated the remarkable increases of *PARP2* and *CASP9* along with the remarkable decreases of *BCL2* and *BCL2L1* associated with *Aβ_1-42_* toxicity. Therefore, the anti-necrotic and antiapoptotic features of the dipeptides cHP1-4, at least in part, are firstly suggested to be responsible for the cyto-protection against *Aβ_1-42_*-induced neurotoxicity by cHP isomers.

The significant increases of AChE and *β*-secretase activities along with a significant reduction of α-secretase activity was observed when SH-SY5Y cells were exposed to *Aβ_1-42_* fragments in this study. These alterations were previously reported by several investigations [40,41,42,43]. However, our results asserted for the first time that cHP1-4 (especially cHP3 and cHP4) slightly modulated AChE activity by *Aβ_1-42_* and cHP1-4 was found as ineffective on *Aβ*-induced alterations on α- and β-secretase activities in the cellular AD model. Thus, it was concluded that cHP1-4 might regulate the transcription for AChE but did not confer to the regulation of *α*- and *β*-secretases. 

The application with *Aβ_1-42_* induced oxidative stress and reduced antioxidant capacity in differentiated SH-SY5Y cells. It was reported that *Aβ*-induced oxidative stress elevated intracellular reactive oxygen species (ROS) levels in the SH-SY5Y cell line [44,45]. The treatment with cHP1-4 (especially cHP4) provided significant positive oxidative alterations via reducing oxidant status and supporting antioxidant capacity in the cellular AD model. As a matter of fact, cHP was shown to protect rat insulinoma cells (RINm5F) from streptozotocin (STZ)-induced in vitro cytotoxicity by minimizing of nitric oxide (NO) production and lipid peroxidation [46]. Furthermore, diketopiperazine ring of the cHP was thought to play a key role in struggling oxidative stress [47]. However, there is scarce data concerned with revealing the antioxidative effect mechanism of cHP. The protective action and its mechanisms were not evaluated yet. Up to now, it was only recorded that cHP activated Nrf2-driven antioxidant response in experimental rats [9]. At this point, our study firstly reports that cHP4 led to remarkable expressional increases of the *SLC7A11* gene that is reduced by *Aβ_1-42_*. SLC7A11 is known as the active component of the cystine/glutamate antiporter complex (system x_c_^−^) that formed by disulfide-linked heterodimerization of SLC3A2 and SLC7A11. System x_c_^−^ contributes to antioxidant defenses via supporting glutathione (GSH) synthesis as well as by ensuring redox balance across the plasma membrane [48,49,50].

The current knowledge on AD pathology suggests that endoplasmic reticulum (ER) dysfunction and unfolded protein response are among the main deterministic of cell death [51]. Here, it was previously reported that ER stress plays an important significant role in *Aβ*-caused cell death in brain endothelial cells; thus, it was proposed that ER stress-targeted therapeutic strategies would be useful for counteracting vascular defects based on neurodegeneration [52,53]. In this context, our results indicated that using cHP1-4 may be a promising strategy for alleviating *Aβ*-induced ER stress. In fact, the treatment with cHP4 led to increases of *ADM2, DNAJB9,* and *UHRF1* gene expressions that were reduced by *Aβ_1-42_* exposure. In supporting this, a significant decrease of *ADM2* gene expression was determined in neural progenitor cells (NPCs) derived from patients with familial AD. Likewise, we found that the expression of *DNAJB9* was downregulated due to *Aβ_1-42_* aggregation in vitro. *UHRF1* gene activity was found to be associated with DNA methylation and posttranslational histone modifications for evading cell death [54,55,56]. *Aβ* also caused impairment of energy homeostasis via leading mitochondrial dysfunctions in the SH-SY5Y cell line [57]. Our findings also revealed that cHP4 reduced the *HSPA1A* expression and increased after *Aβ_1-42_* exposure. *HSPA1A* level was increased after treatment with *Aβ_1-42_* due to the accumulation of misfolded protein molecules [58]. Besides, cHP4 modulated the expressional change of the gene *ACADVL* reduced by *Aβ_1-42_* that has prominent functions in the mitochondria and is required for fatty acid oxidation.

A previous study by Bellezza et al. [9] proved that cHP inhibited the pro-inflammatory NF-κB pathway. Due to this anti-inflammatory role of cHP, this dipeptide has been tested against neuropathological conditions. Our results determined that cHP4 provides an ameliorating potential via elevating *METAP2* expression decreased by *Aβ_1-42_*. *METAP2* is reported to be responsible for promoting cell proliferation in SH-SY5Y neuroblastoma cells and for exhibiting immune-modulatory activity [59,60]. On the other hand, it is known that *Aβs* (such *Aβ_1-42_* and *Aβ_25-35_*) induced DNA damages via enhancing 8-OH-dG adduct levels, numbers of apurinic/apyrimidinic sites, and both DNA single or double-stranded breaks rat cortical neuron cultures [61,62,63]. In this context, our molecular genetic analysis revealed that cHP4 reduced the *ERCC1* gene expression that was increased due to DNA damage by *Aβ_1-42_*. The *ERCC1* gene is known to play a key function in the nucleotide excision repair (NER) pathway and is critically required for the DNA repair process [64]. Thus, it is concluded that the observed neuroprotection mechanism of cHP4 is also associated with a contribution to DNA repair mechanisms. 

In this study, we performed different cytotoxicity and genotoxicity assays to reveal the safety and toxicological profiling of the most effective isomer, cHP4. MTT and LDH release assays on cultured whole human blood cells showed the noncytotoxic property of cHP4. Further, genotoxicity testing using SCE, MN, and 8-OH-dG assays determined that cHP4 is also non-genotoxic. In this context, the evaluation of toxicological aspects of cHP isomers, especially cHP4, was considered as essential due to their potential use as promising drug candidates. In fact, eptastigmine, a previously developed drug, is found to cross the blood brain barrier (BBB) easily and to be very effective in inhibiting AChE activity. However, its usage in clinical trials was terminated due to its hematotoxicity action [65].

The present study revealed that cHP1-4 provided a different degree of neuroprotection against *Aβ_1-42_* exposure. cHP1-4 alleviated *Aβ_1-42_*-induced necrotic and apoptotic cell death and oxidative alterations. Different in vivo studies indicated that Aβ pathology accounts for the cognitive dysfunction in AD via instabilities in neuronal activities but that Aβ-induced memory problems alone cannot be sufficient to explain all symptoms of AD patients [66]. Although transgenic mouse models were constituted to overexpress proteins linked to mutant amyloid precursor protein (APP), familial AD (FAD), and presenilin (PS) for exhibiting AD pathology, the overexpressed gene-related interactions caused other phenotypes unrelated to AD [67]. Also, an in vivo model constructed expressing APP can act differently from human APP because the APP gene sequence shows some dissimilarities from a murine sequence. Thus, it is impossible to stimulate the exact same AD pathology as the human by using mice or rat models [68]. By these points, in vitro human cell culture models related to AD would be appropriate to stimulate closer toxicological phenotypes with AD patients for preclinical studies. The isomers slightly modulated the AChE activity but not α- and β-secretase activities that were altered by *Aβ.* Previous studies investigated that CHP is ubiquitous in the Cerebrospinal fluid (CSF) and has important roles in pain awareness, body core temperature, food intake, and modulating prolactin secretion acting as an endocrine effector. When the common point of these actions was analyzed, dopaminergic mechanisms were found to be the main player of these systems. It was reported that the increase of CHP-like compounds in CSF can improve cognitive function and can enhance neurological recovery after trauma [69]. The cHP4 was determined as the most effective isomer among cHP1-4. Moreover, the underlying molecular mechanisms of neuroprotection by cHP4 was found to be related to (I) apoptosis and necrosis, (II) DNA repair, (III) oxidative stress, (IV) ER stress and unfolded protein response, (V) mitochondrial energy metabolism, and (VI) immunity pathways. Besides, the isomer exhibited noncytotoxic and non-mutagenic features on cultured human blood cells. Future in vivo research is required to further explore the neuroprotective actions of cHP1-4 for the development of an effective treatment strategy against AD.

## Figures and Tables

**Table 1:** The effects of cHP1-4 on Aβ1-42-induced cell death. 3-(4,5-dimethylthiazol-2-yl)-2,5-diphenyltetrazolium bromide (MTT) and lactate dehydrogenase (LDH) release tests were used to identify cell viabilities in each group.

Groups	% Cell Viability (MTT Assay)	% Cell Viability (LDH Assay)
Control (−)	100 ^e^	100 ^g^
20 µM *Aβ_1-42_*	50.8 ± 3.7 ^a^	48.9 ± 2.5 ^a^
*Aβ +* 0.1 µM cHP1	50.9 ± 3.2 ^a^	48.7 ± 2.7 ^a^
*Aβ +* 1 µM cHP1	51.3 ± 4.0 ^a^	49.5 ± 3.0 ^a^
*Aβ +* 10 µM cHP1	52.5 ± 3.7 ^a^	50.9 ± 2.5 ^a^
*Aβ +* 25 µM cHP1	56.2 ± 4.4 ^ab^	53.6 ± 2.4 ^ab^
*Aβ +* 50 µM cHP1	56.8 ± 3.5 ^ab^	57.1 ± 2.8 ^b^
*Aβ +* 100 µM cHP1	57.9 ± 4.1 ^ab^	58.8 ± 3.0 ^b^
*Aβ +* 0.1 µM cHP2	51.5 ± 3.3 ^a^	49.9 ± 2.8 ^a^
*Aβ +* 1 µM cHP2	52.7 ± 4.2 ^a^	50.6 ± 3.1 ^a^
*Aβ +* 10 µM cHP2	55.8 ± 5.0 ^ab^	52.9 ± 2.7 ^a^
*Aβ +* 25 µM cHP2	57.1 ± 4.8 ^ab^	53.1 ± 2.8 ^ab^
*Aβ +* 50 µM cHP2	56.9 ± 4.1 ^ab^	58.7 ± 2.4 ^b^
*Aβ +* 100 µM cHP2	59.7 ± 3.8 ^b^	60.1 ± 3.3 ^b^
*Aβ +* 0.1 µM cHP3	51.7 ± 4.1 ^a^	53.5 ± 3.0 ^ab^
*Aβ +* 1 µM cHP3	55.5 ± 4.3 ^ab^	58.9 ± 2.5 ^b^
*Aβ +* 10 µM cHP3	59.6 ± 4.7 ^b^	63.6 ± 2.8 ^bc^
*Aβ +* 25 µM cHP3	66.5 ± 4.6 ^b^	66.8 ± 3.0 ^c^
*Aβ +* 50 µM cHP3	69.0 ± 5.0 ^b^	72.4 ± 3.3 ^d^
*Aβ +* 100 µM cHP3	71.3 ± 6.6 ^bc^	77.3 ± 3.5 ^de^
*Aβ +* 0.1 µM cHP4	52.8 ± 3.6 ^a^	52.4 ± 2.7 ^ab^
*Aβ +* 1 µM cHP4	55.9 ± 4.7 ^ab^	56.8 ± 2.9 ^b^
*Aβ +* 10 µM cHP4	63.9 ± 5.2 ^b^	67.0 ± 3.4 ^c^
*Aβ +* 25 µM cHP4	71.2 ± 6.8 ^bc^	74.6 ± 3.2 ^d^
*Aβ +* 50 µM cHP4	79.4 ± 6.2 ^c^	82.3 ± 3.5 ^e^
*Aβ +* 100 µM cHP4	87.9 ± 6.6 ^d^	88.5 ± 3.6 ^f^

Means with a different letter in the same column are significantly different from each other at the level of *p* < 0.05.

**Table 2:** The observed total antioxidant capacity (TAC) and total oxidative status (TOS) levels in cultured differentiated SH-SY5Y cells after treatment with cHP1-4.

Groups	TAC Level (mmolTrolox Equiv./L)	TOS Level (µmol H_2_O_2_ Equiv./L)
Control (−)	4.8 ± 0.7 ^b^	2.1 ± 0.3 ^a^
Control (+)	12.7 ± 1.3 ^f^	5.7 ± 0.6 ^c^
20 µM *Aβ_1-42_*	2.6 ± 0.4 ^a^	5.1 ± 0.5 ^b^
0.1 µM cHP1	4.9 ± 0.6 ^b^	2.0 ± 0.3 ^a^
1 µM cHP1	5.2 ± 0.8 ^b^	1.8 ± 0.2 ^a^
10 µM cHP1	5.6 ± 0.7 ^c^	2.0 ± 0.3 ^a^
25 µM cHP1	6.1 ± 0.6 ^c^	2.3 ± 0.4 ^a^
50 µM cHP1	6.3 ± 0.5 ^c^	2.2 ± 0.2 ^a^
100 µM cHP1	6.5 ± 0.7 ^c^	2.0 ± 0.2 ^a^
0.1 µM cHP2	4.7 ± 0.5 ^b^	1.7 ± 0.1 ^a^
1 µM cHP2	4.9 ± 0.7 ^b^	2.0 ± 0.2 ^a^
10 µM cHP2	5.3 ± 0.6 ^b^	2.1 ± 0.2 ^a^
25 µM cHP2	5.4 ± 0.8 ^b^	2.3 ± 0.3 ^a^
50 µM cHP2	6.1 ± 0.6 ^c^	2.3 ± 0.4 ^a^
100 µM cHP2	6.4 ± 0.7 ^c^	2.4 ± 0.3 ^a^
0.1 µM cHP3	5.0 ± 0.3 ^b^	1.9 ± 0.2 ^a^
1 µM cHP3	5.3 ± 0.5 ^c^	2.0 ± 0.2 ^a^
10 µM cHP3	5.8 ± 0.4 ^c^	2.0 ± 0.2 ^a^
25 µM cHP3	6.7 ± 0.5 ^c^	2.2 ± 0.3 ^a^
50 µM cHP3	7.1 ± 0.8 ^d^	2.2 ± 0.3 ^a^
100 µM cHP3	7.6 ± 0.5 ^d^	2.4 ± 0.2 ^a^
0.1 µM cHP4	4.9 ± 0.7 ^b^	1.8 ± 0.1 ^a^
1 µM cHP4	5.5 ± 0.7^b^	2.0 ± 0.3 ^a^
10 µM cHP4	6.4 ± 0.6 ^c^	2.0 ± 0.2 ^a^
25 µM cHP4	6.8 ± 0.4 ^c^	2.0 ± 0.2 ^a^
50 µM cHP4	7.7 ± 0.7 ^d^	1.9 ± 0.1 ^a^
100 µM cHP4	8.9 ± 0.9 ^e^	2.0 ± 0.2 ^a^

Means with a different letter in the same column are significantly different from each other at level of *p* < 0.05.

**Table 3:** The observed TAC and TOS levels in cultured differentiated SH-SY5Y cells after simultaneously treatment with cHP1-4 and Aβ1-42.

Groups		TAC Level (mmolTrolox Equiv./L)	TOS Level (µmol H_2_O_2_ Equiv./L)
Control (−)		4.8 ± 0.7 ^d^	2.1 ± 0.3 ^a^
Control (+)		12.7 ± 1.3 ^e^	5.7 ± 0.6 ^e^
20 µM *Aβ_1-42_*		2.6 ± 0.4 ^a^	5.1 ± 0.5 ^d^
*Aβ_1-42_* plus cHP1	0.1 µM	2.5 ± 0.5 ^a^	5.0 ± 0.7 ^d^
	1 µM	2.6 ± 0.4 ^a^	4.8 ± 0.5 ^d^
	10 µM	2.7 ± 0.6 ^a^	4.7 ± 0.5 ^d^
	25 µM	2.8 ± 0.5 ^a^	4.4 ± 0.6 ^d^
	50 µM	3.0 ± 0.7 ^ab^	4.0 ± 0.5 ^c^
	100 µM	3.2 ± 0.8 ^ab^	3.9 ± 0.4 ^c^
*Aβ_1-42_* plus cHP2	0.1 µM	2.7 ± 0.5 ^a^	4.8 ± 0.6 ^d^
	1 µM	2.8 ± 0.5 ^a^	4.7 ± 0.7 ^d^
	10 µM	2.8 ± 0.5 ^a^	4.5 ± 0.5 ^d^
	25 µM	3.0 ± 0.7 ^ab^	4.4 ± 0.4 ^d^
	50 µM	3.1 ± 0.7 ^ab^	4.3 ± 0.6 ^d^
	100 µM	3.2 ± 0.8 ^ab^	4.0 ± 0.4 ^c^
*Aβ_1-42_* plus cHP3	0.1 µM	2.7 ± 0.6 ^a^	4.7 ± 0.3 ^d^
	1 µM	2.9 ± 0.5 ^a^	4.6 ± 0.4 ^d^
	10 µM	3.1 ± 0.7 ^ab^	4.5 ± 0.5 ^d^
	25 µM	3.4 ± 0.7 ^b^	4.2 ± 0.4 ^d^
	50 µM	3.5 ± 0.6 ^b^	3.9 ± 0.6 ^c^
	100 µM	3.8 ± 0.8 ^c^	3.6 ± 0.5 ^c^
*Aβ_1-42_* plus cHP4	0.1 µM	2.7 ± 0.6 ^a^	4.6 ± 0.7 ^d^
	1 µM	2.9 ± 0.5 ^a^	4.3 ± 0.6 ^d^
	10 µM	3.5 ± 0.7 ^b^	4.0 ± 0.5 ^c^
	25 µM	3.7 ± 0.6 ^b^	3.6 ± 0.4 ^c^
	50 µM	3.9 ± 0.8 ^c^	3.0 ± 0.4 ^b^
	100 µM	4.6 ± 1.0 ^d^	2.7 ± 0.3 ^b^

Means with a different letter in the same column are significantly different from each other at level of *p* < 0.05.

**Table 4:** The gene expression alterations (as fold change).

Gene	*Aβ_1-42_*	*Aβ_1-42_* plus CHP4
*ACADVL*	−0.77	3.83
*AKT1*	0.15	0.08
*ADM2*	−0.43	3.68
*BCL2*	−0.94	4.67
*BCL2L1*	−0.77	5.12
*CASP8*	3.45	2.89
*CASP9*	2.91	0.83
*CYP2D6*	−0.45	1.75
*DNAJB9*	−0.63	2.35
*ERCC1*	10.25	2.55
*FASLG*	1.14	0.64
*HSPA1A*	3.66	0.16
*METAP2*	−0.71	3.13
*PARP2*	7.93	2.06
*SLC7A11*	−0.38	13.14
*UHRF1*	−0.54	3.12

**Table 5:** The effects of cHP4 applications (0.1-100 µM) on the frequencies of SCE/cell and MN/2000 cell as well as the rate of NDI on cultured human lymphocyte cells.

Groups		SCEs/Cell	MN/2000 Cells	Nuclear Division Index (NDI)
Control (-)		4.6 ± 0.7 ^a^	0.8 ± 0.1 ^a^	1.5 ± 0.3 ^b^
Control (+)		11.4 ± 0.9 ^b^	3.9 ± 0.5 ^b^	1.0 ± 0.2 ^a^
cHP4	0.1 µM	4.3 ± 0.4 ^a^	0.7 ± 0.2 ^a^	1.4 ± 0.2 ^b^
	1 µM	4.1 ± 0.7 ^a^	0.8 ± 0.2 ^a^	1.4 ± 0.1 ^b^
	10 µM	4.5 ± 0.5 ^a^	0.9 ± 0.2 ^a^	1.4 ± 0.2 ^b^
	25 µM	4.3 ± 0.7 ^a^	0.8 ± 0.2 ^a^	1.4 ± 0.3 ^b^
	50 µM	4.7 ± 0.6 ^a^	0.9 ± 0.2 ^a^	1.3 ± 0.2 ^b^
	100 µM	4.6 ± 0.5 ^a^	0.8 ± 0.2 ^a^	1.3 ± 0.2 ^b^

Means with the different letter in the same column are significantly different from each other at level of *p* < 0.05.

## Supplementary Materials

The following are available online at https://www.mdpi.com/2218-273X/10/5/737/s1, Figure S1: Cyclo(His-Pro) isomers (cHP1-4), Figure S2: (a) Undifferentiated and (b) differentiated SH-SY5Y via treating with RA, Table S1: Cell cycle distribution of SH-SY5Y cells treated with all-trans retinoic for 11 days as determined by flow cytometry.
