# Supplementary material for: Histidyl-Proline Diketopiperazine Isomers as Multipotent Anti-Alzheimer Drug Candidates
Source: Biomolecules. 2020 May 9;10(5):737. doi: 10.3390/biom10050737 (PMC7277666; doi:10.3390/biom10050737)

biomolecules

Supplementary Materials

Histidyl-Proline Diketopiperazine Isomers as Multipotent Anti-Alzheimer Drug Candidates

Hasan Turkez[1,*], Ivana Cacciatore[2], M. Enes Aslan[3], Erika Fornasari[2], Lisa Marinelli[2], Antonio Di Stefano[2], Adil Mardinoglu[4,5,*]

[1] Department of Medical Biology, Faculty of Medicine, Atatürk University, Erzurum, Turkey.
[2] Department of Pharmacy, University "G. d'Annunzio" of Chieti-Pescara, via dei Vestini 31, 66100 Chieti Scalo (CH), Italy.
[3] Department of Molecular Biology and Genetics, Faculty of Science, Erzurum Technical University, Erzurum, Turkey.
[4] Science for Life Laboratory, KTH-Royal Institute of Technology, Stockholm, SE-17121, Sweden.
[5] Centre for Host-Microbiome Interactions, Faculty of Dentistry, Oral & Craniofacial Sciences, King's College London, London, SE1 9RT, United Kingdom.

* Corresponding authors: Prof. Dr. Adil Mardinoglu (e-mail: adilm@scilifelab.se) and Prof. Dr. Hasan Turkez (hturkez@atauni.edu.tr)

Table S1. Cell cycle distribution of SH-SY5Y cells treated with all-trans retinoic for 11 days as determined by flow cytometry.

	Cell Population (%)			
**Group**	**G1 phase**	**G2 phase**	**S phase**	**G2/G1**
Control	50.18 ± 3.12	13.65 ± 0.89	34.74 ± 1.54	1.43 ± 0.09
RA treated	76.39 ± 3.24*	2.45 ± 0.12*	19.4 3± 1.81*	1.86 ± 0.11

Values are expressed as the mean ± standard deviation. Symbol (*) represents statistically significant difference ($p < 0.05$) compared with control.

Figure S1. Cyclo(His-Pro) isomers (**cHP1-4**).

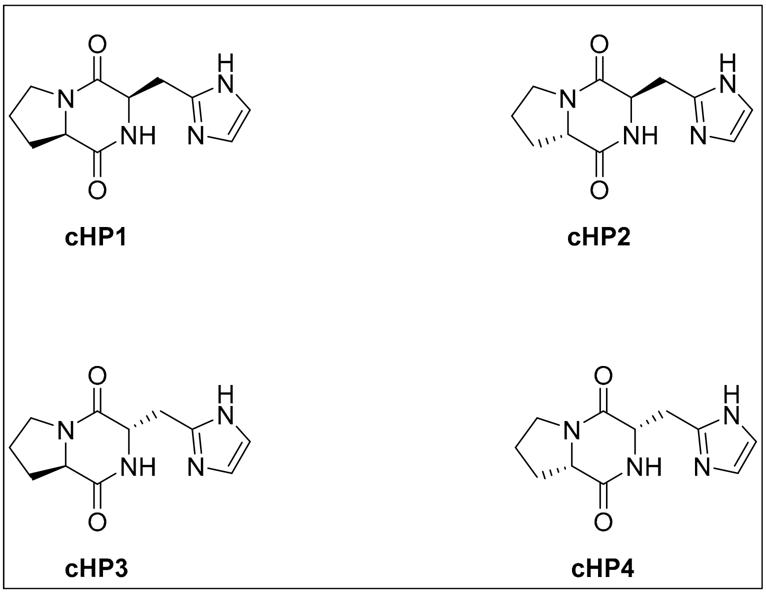

Figure S2. (**a**) Undifferentiated and (**b**) differentiated SH-SY5Y via treating with RA.

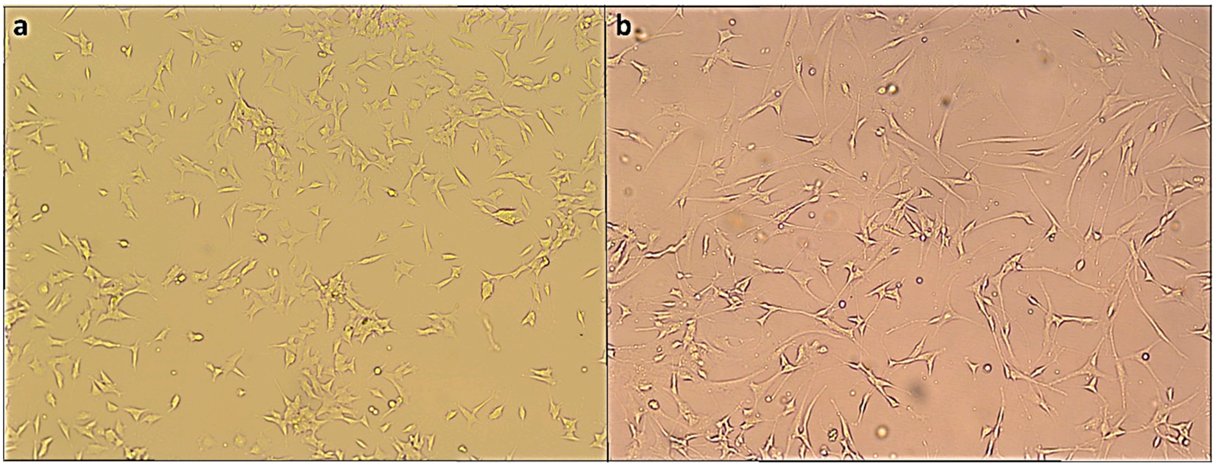

Supplement: Supplementary file 1 [file biomolecules-10-00737-s001.pdf]
